# Abdominal X-Ray Findings of Patients With Crohn’s Disease Presenting With Acute Abdominal Pain

**DOI:** 10.7759/cureus.32484

**Published:** 2022-12-13

**Authors:** Nouran W Molla, Abdullah H Alsergani, Nouf Alharbi, Fahad A Alfaiz, Abduljabbar A Alyamani, Abdulaziz A Aljohani, Majed A AlJohani, Mohammed BinMayouf

**Affiliations:** 1 Radiology, King Saud University Medical City, Riyadh, SAU; 2 Medicine, King Saud University Medical City, Riyadh, SAU; 3 Radiology, King Saud University, Riyadh, SAU; 4 Medicine and Surgery, King Khalid University Hospital, Riyadh, SAU; 5 Medicine, King Saud University, Riyadh, SAU; 6 Dermatology, King Khalid University Hospital, Riyadh, SAU

**Keywords:** saudi arabia, radiation risk, acute complications of crohn's disease, crohn's disease exacerbation, inflammatory bowel disease, x-ray findings, plan film of the abdomen, abdominal x-ray, acute abdominal pain, crohn’s disease (cd)

## Abstract

Objectives: The primary objective of this study is to describe the most common radiological findings found on abdominal X-rays of patients with Crohn’s disease (CD) presenting with acute abdominal pain.

Methods: A cross-sectional study was conducted at a tertiary care hospital in Riyadh. Data from CD patients who presented with acute abdominal pain between December 2016 and December 2021 was analyzed. A total of 144 abdominal X-rays met the inclusion and exclusion criteria and were included in the study. The medical records of patients who had the X-rays were subsequently reviewed for the presence or absence of follow-up imaging studies.

Results: Of the 144 abdominal X-ray studies, 54 (37.5%) had positive findings, while 90 (62.5%) were unremarkable. The most common category of findings was small bowel findings (32.6%), acute complications (32.6%), followed by extraintestinal findings (2.7%), and colonic findings (1.35%). About 29.2% of the abdominal X-rays had subsequent follow-up imaging done. The multivariate logistic binary regression analysis demonstrated that males had an odds ratio of 2.25 of undergoing follow-up imaging compared to females (p = 0.049).

Conclusion: The non-specific findings found on the majority of the abdominal X-rays may indicate that it is of limited diagnostic value in this patient population. However, they play an integral role in ruling out acute complications in CD patients presenting with abdominal pain and exhibiting disease activity.

## Introduction

Crohn’s disease (CD) is a systemic relapsing and remitting inflammatory bowel disease (IBD) that affects any segment of the gastrointestinal tract. Patients with CD might present with abdominal pain, fever, diarrhea, or signs of bowel obstruction. They may also present with a number of extraintestinal symptoms such as erythema nodosum or ankylosing spondylitis [1]. The incidence of CD has been shown to be three to 20 cases per 100,000. It is more common in developed countries, particularly in North America and Western Europe. Its incidence in women was found to be more than in men and has been linked to people of Ashkenazi Jewish descent [2]. CD is thought to be of multifactorial origin and has been linked to an interplay between genetic susceptibility, environmental factors, and an alteration in the intestinal flora. These factors are thought to contribute to an abnormal immune response in the gut, which compromises its mucosal epithelial defensive function [3].

The management of CD varies significantly between patients. Medical management of CD involves the usage of steroids, monoclonal antibody therapies, and immunomodulators. Surgery is usually reserved for patients who have developed severe complications or are refractory to medical therapy [4]. CD can lead to many complications such as fibro-stenotic strictures, fistulas, and abscess formation. Besides these complications, it is linked to numerous intestinal and extraintestinal malignancies such as colorectal carcinoma (CRC) and T-cell lymphoma [3]. The relative risk (RR) of CD patients to develop small bowel malignancies compared to a normal population was found to be 28.4 according to a systematic review [5].

Imaging plays an integral role in CD. It has a role in the primary diagnosis of the disease, the monitoring of disease activity, and the assessment of complications. Different imaging modalities can be used over the course of a patient’s disease. Examples of imaging modalities used in CD patients are abdominal X-rays, small bowel follow-through, computed tomography enterography (CTE), magnetic resonance enterography (MRE), and transabdominal ultrasound [6]. CTE and MRE were reported to have a sensitivity of over 95% for the detection of CD, though the gold standard for the diagnosis of Crohn’s disease remains to be tissue biopsy [7].

A study conducted by Al-Ghamdi et al. measured the incidence of CD in Saudi Arabia between 1983 and 2002. They found that the average annual incidence of the CD over the first 10 years was 0.32:100,000 and 1.66:100,000 over the latter 10 years. The exponential increase in incidence may indicate the upward trend of the disease in Saudi Arabia [8]. This has driven clinicians to research different aspects of the disease in order to improve the approach and management of patients with CD in the region. However, there are currently no studies that assess the effectiveness of abdominal X-rays for CD patients presenting with acute abdominal pain. The primary objective of this study is to describe the most common radiological findings found on abdominal X-rays of patients with CD presenting with acute abdominal pain.

## Materials and methods

Study design

**Figure 1:**
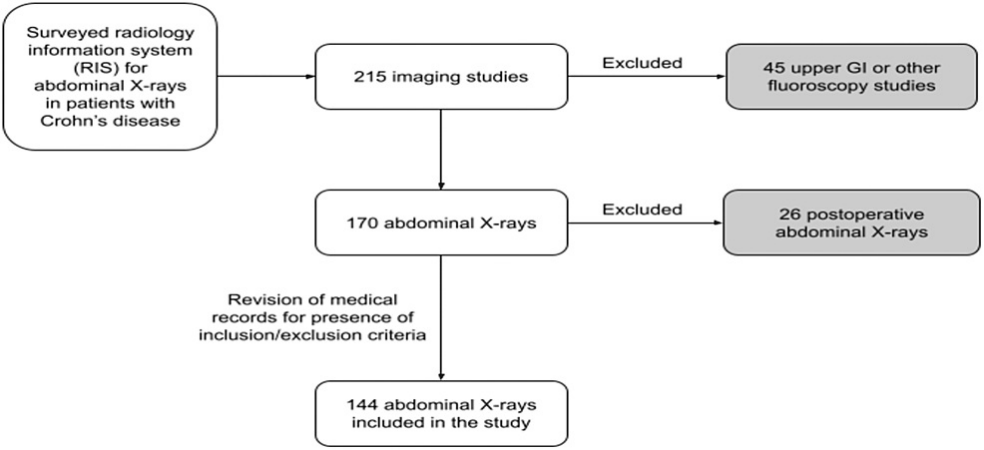
Flowchart demonstrating the sample selection process

This is a cross-sectional study that was done at a tertiary care center in Riyadh, Saudi Arabia. The required data was gathered using E-SIHI (Electronic System for Integrated Health Information), PACS (Picture Archiving and Communication System), and the RIS (Radiology Information System). The inclusion criteria of this study were (1) patients who are above 18 years old, (2) patients who have a pathology-proven diagnosis of CD, and (3) patients who had an abdominal X-ray taken upon presenting with acute abdominal pain. Acute abdominal pain was defined as any feeling of constant pain in the abdominal area felt by the patient within five days of presentation to the hospital. The exclusion criterion was any postoperative abdominal X-ray within 10 days of surgery. All abdominal X-rays were obtained in the anteroposterior (AP) projection while the patient was in the supine position. After obtaining ethical approval from the hospital's IRB (Project No.: E-22-6584), imaging studies of patients with CD presenting with acute abdominal pain were gathered and subsequently reviewed between December 2016 and December 2021. A total of 215 studies were collected. After applying the inclusion and exclusion criteria, only 144 abdominal X-rays were included in the cohort of this study as 45 were excluded for the lack of plain abdominal X-rays at the time of presentation (upper GI studies or other fluoroscopy studies) and 26 studies were excluded for being postoperative images as demonstrated by Figure 1.

Demographic data at the time of presentation including age and gender were collected. The reported X-ray findings were divided into four categories. The categories were small bowel findings, colonic findings, acute complications, and extraintestinal manifestations. The four categories were subdivided into multiple subsections. The subsections were small bowel mucosal edema, dilated small bowel loops, displaced small bowel loops under small bowel findings, colonic mucosal edema, colonic dilatation, toxic megacolon, pneumatosis coli under colonic findings, pneumoperitoneum, abscess collection, obstruction under acute complications, and extraintestinal findings. The abdominal X-rays were then read and analyzed by a radiology resident of the institute; this study was conducted and further reviewed by an abdominal imaging consultant for the presence or absence of any of the findings mentioned above. The presence of follow-up imaging was assessed through the patient’s records. Follow-up imaging was defined as any additional imaging that was done within five days of the original image. This included CT scan, MRI, and other abdominal X-ray images.

Statistical analysis

The mean and standard deviation were used to describe continuous metric variables, while the frequencies and percentages were used for the categorically measured variables. The statistical normality assumption was tested via the Kolmogorov-Smirnov test. The multiple response dichotomies analysis was used to describe the patient's abdominal X-ray’s main findings. The multivariate logistic regression analysis was used to assess the associations between the patients' age, gender, and X-ray findings with their odds of requiring follow-up imaging. The associations between the predictors in the logistic regression with their dependent outcome variable were expressed as odds ratios (ORs) with their associated 95% confidence interval (CI). SPSS version 21 (IBM Corp., Armonk, NY), the commercially available statistical data analysis program, was used for the statistical data analysis, and the statistical significance was considered at 0.05 level.

## Results

A retrospective review of 144 abdominal X-rays of patients diagnosed with CD who presented with acute abdominal pain was conducted. Table 1 displays the yielded descriptive analysis findings for the patients’ sociodemographic characteristics; 57.6% of the patients were males and 42.4% were females. The mean ± SD age in years for the sample of patients was equal to 29.92 ± 10.77 years. Most of them (45.1%) were aged between 18 and 25 years, and 31.9% were aged between 26 and 35 years; 13.9% of the patients were aged ≥ 46 years, while the remainder (9%) were aged between 36 and 45 years.

**Table 1:** Descriptive analysis of the patient’s sociodemographic characteristics (N = 144)

	Frequency	Percentage
Sex		
Female	61	42.4
Male	83	57.6
Age (years), mean (SD)		29.92 (10.77)
Age group		
18-25 years	65	45.1
26-35 years	46	31.9
36-45 years	13	9
≥46 years	20	13.9

Table 2 displays the yielded descriptive analysis of the patients’ abdominal X-ray findings. The resulting findings showed that there were a total of 152 significant findings that were revealed on 54 positive abdominal X-rays out of 144 (37.5%). The remainder of the X-rays (N = 90) were normal. The main findings of the positive abdominal X-rays were as follows: 87% of the positive abdominal X-rays had small bowel findings, 87% of them showed acute intestinal complications, 7.4% of them had extraintestinal findings, and 3.7% of them had colonic findings. Concerning the small bowel findings, 32.6% of the patients had dilated small bowel loops. Regarding the main colonic findings, the resulting analysis showed that only two patients demonstrated large bowel changes (colonic dilations and colonic mucosal edema), while the remaining (98.6%) of the patients had no major large bowel findings upon revision of their abdominal X-rays. Regarding acute intestinal complications, 31.9% showed evidence of small bowel ileus or obstructions, while 0.7% of the patients showed positive evidence for abdominal abscess or fluid collections. Moreover, concerning the extraintestinal main findings, two patients showed evidence of gallstones, while another two showed evidence of renal tract calculi. The remaining majority of the patients had no extraintestinal-positive findings on their abdominal X-rays. In general, the resulting findings from the analysis showed that 36.1% of the abdominal X-rays had positive evidence of significant findings, and 63.9% of them were unremarkable. Upon revising the medical records, 29.2% of the abdominal X-rays had subsequent follow-up imaging within five days.

**Table 2:** Descriptive analysis of the patient’s abdominal X-ray findings and outcomes (N = 144) SB: Small bowel.

Summary of main revealing X-ray findings (N = 54)	Frequency	Percentage of presence within abnormal X-ray
Small bowl findings	47	87
Colonic findings	2	3.7
Acute complications	47	87
Extraintestinal findings	4	7.4
Small bowel findings		
Dilated SB loops	47	32.6
None	97	67.4
Large bowel findings		
Colonic dilatation	1	0.7
Colonic mucosal edema	1	0.7
None	142	98.6
Acute complications		
Collection or abscess	1	0.7
SB ileus or obstruction	46	31.9
None	97	67.4
Extraintestinal findings		
Gallstones	2	1.4
Renal tract calculi	2	1.4
None	140	97.2
Required follow-up imaging		
No	102	70.8
Yes	42	29.2

The multivariate logistic binary regression analysis was conducted to assess what available demographic findings may explain the patients’ need for further follow-up imaging after presenting with acute abdominal pain as displayed in Table 3. The iterative and interim analysis models showed that the abdominal findings did not correlate significantly with the patients' odds of requiring follow-up imaging post presenting with acute abdominal pain. The patients’ age also did not correlate significantly with their odds of requiring more follow-up imaging (p = 0.300). Although the patients’ age in years showed a slightly positive correlation with the need for follow-up imaging, the analysis model suggested that males were predicted to be significantly more inclined to require follow-up imaging (2.25 times more) compared to female patients when presenting with acute abdominal complaints, given their abdominal X-ray abnormalities (p-value = 0.049).

**Table 3:** Multivariate adjusted association between the patients' age and sex with their need for follow-up imaging when presenting with acute abdominal pain Dependent variable means requiring further follow-up imaging (No/Yes) after presenting with acute abdominal complaints.

	Multivariate adjusted (OR)	95% CI for OR		p-values
		Lower	Upper	
Age (years)	1.019	.984	1.055	.300
Gender = Male	2.253	1.005	5.050	.049
Constant	.143			.006

## Discussion

The primary objective of this study is to describe the most common radiological findings found on abdominal X-rays of patients with CD presenting with acute abdominal pain. The majority of patients included in this study were between the ages of 18 and 25 (45.1%), followed by patients between 26 and 35 (31.9%), patients above the age of 45 (13.9%), and patients between 36 and 45 (9%).

In this study, approximately two-thirds of the abdominal X-rays were normal. Acute complications and small bowel findings were the most common abnormalities. The most common acute complication was ileus/obstruction. Only one abdominal collection/abscess was detected. Small bowel findings included small bowel dilation, which are relatively non-specific findings in patients with known CD. Nearly one-third of patients required follow-up imaging. The aforementioned small bowel findings are consistent with a previous study by O’Regan et al. assessing the value of plain abdominal radiographs in 399 patients with CD [9]. Although the frequency of acute complications was found to be interestingly significantly higher in this study, it is likely because this study only involved patients who were actively presenting with abdominal pain and exhibited disease flare-up as opposed to their study that did not have this specific inclusion criterion. O’Regan et al. also found that despite abdominal X-rays being the most commonly ordered study (approximately one-third of all imaging studies in CD patients), over two-thirds of them were normal, with nearly 40% of them needing additional imaging within five days. Only one percent of the performed abdominal X-rays displayed significant complications of CD [9]. Although this study demonstrated that many of the findings on the X-rays were non-specific, the value of the X-rays might lie in ruling out acute complications that necessitate escalation of medical management. The results of this study showed that 52 out of 144 abdominal X-rays exhibited acute complications. This is consistent with a previous study that found abdominal X-rays to have a sensitivity as high as 76% for CD in children [10]. Although they were not relied on for diagnosis, radiological studies such as small bowel follow-through, CTE, and MRE are more valuable modalities [6]. Despite limited diagnostic value, abdominal X-rays are usually indicated as the first-line imaging modality in patients who are known cases of CD who present with new-onset abdominal pain to rule out acute complications. They can also be used to assess small bowel findings, colonic findings, and extraintestinal findings [9].

In addition, the results of this study indicated that male patients were twice as likely to undergo follow-up imaging than females, although no significant differences in specific findings were noted. The explanation for this finding may be because males generally have a more severe course of CD than females as shown by multiple studies [11,12], the latter of which is a cohort study following 260 patients with CD over the course of 12 years. Apart from the differences in disease courses, they also found that male patients were twice as likely to have acute complications. Another study from Mayo Clinic concluded that males were more likely to undergo abdominal surgery due to complications of the disease [13].

Patients with IBD are at an increased risk of developing many malignancies including colorectal cancer, small bowel adenocarcinoma, and cholangiocarcinoma [14]. There is clear evidence demonstrating an increased cancer risk when radiation exposure exceeds 50 millisieverts (mSv) [15]. Each abdominal X-ray exposes a patient to 1.2 mSv, while an abdominal CT scan exposes them to 10 mSv [16]. According to the meta-analysis by Chatu et al., approximately 11% of patients with CD are exposed to a dose of radiation exceeding 50 mSv [17]. Exposing patients with CD to excess abdominal X-rays may be exposing them to unnecessary additional radiation with low diagnostic yield, especially considering that nearly one-third of them need follow-up imaging. In addition to the unnecessary additional exposure to radiation, the additional incurred cost of these studies must be considered. CD already represents a heavy financial burden. The cost of the administration of the most commonly used biologic (infliximab) alone is nearly 8000 dollars per year [18].

This study is significant for showcasing the potential value of abdominal X-rays in this patient population. It also provides a baseline for the pattern of findings that clinicians can expect to find on abdominal X-rays of CD patients presenting with abdominal pain. This needs to be considered before ordering them excessively and without clear indications, given the radiation risks and increased costs. Despite this study's potential significance, it has limitations such as the small sample size of 144 abdominal X-rays.

## Conclusions

CD is a debilitating illness associated with many physical, emotional, and financial burdens. It is a disease with many potential complications that require urgent medical attention. This study showed that approximately one-third of all abdominal X-rays done on patients with CD presenting with abdominal pain had positive findings. Of these, the most common categories were small bowel findings, followed by acute complications. The study also showed that about a third of the patients had undergone follow-up imaging within five days of the initial abdominal X-ray. Given these conclusions, clinicians should carefully weigh the risks and benefits of ordering abdominal X-rays on this patient population. They were shown to be important for ruling out acute complications but had low diagnostic yield otherwise. More studies targeting this exact topic are needed to reach more concrete conclusions.
